# Breast cancer-associated opsoclonus-myoclonus syndrome: a case report

**DOI:** 10.1186/s12957-021-02436-7

**Published:** 2021-11-16

**Authors:** Aikaterini Kostoglou, Dimitrios Vlastos, Athanasios Bakalis, Debashis Ghosh

**Affiliations:** 1 Department of Surgery, University College London, Royal Free London NHS Foundation Trust, London, UK; 2 Department of Cardiac Surgery, Liverpool Heart and Chest Hospital, Liverpool, UK

**Keywords:** Paraneoplastic syndrome, Breast cancer, Anti-Ri, Opsoclonus-myoclonus syndrome

## Abstract

**Background:**

Paraneoplastic neurological syndromes constitute rare neurological complications of malignant disease, manifesting in <1% of patients with cancer. Opsoclonus-myoclonus syndrome (OMS) presents with chaotic ocular saccades (opsoclonus), spontaneous muscular jerking (myoclonus) that may be accompanied by ataxia, strabismus, aphasia, or mutism. Its paraneoplastic variant in the adult is most commonly associated with small-cell lung cancer, followed by breast cancer. Importantly, neurological symptoms usually precede the diagnosis of breast cancer and tend to recure after its treatment.

**Case presentation:**

A 43-year-old premenopausal Caucasian woman with a medical history of hypertension was admitted following an episode of focal seizure. This progressed to generalised tonic-clonic seizures and she was subsequently loaded with phenytoin, valproate, and levetiracetam. Initial workup included whole body CT scan, viral and autoimmune serology. The CT scan revealed an enhancing right axillary lymph node, which in combination with Anti-Ri antibody positivity raised the spectre of paraneoplastic OMS. MRI of the head revealed subtle nonspecific white matter signal change within the centrum semiovale without any mass lesions, while MRI of the spine was unremarkable. An uncomplicated right mastectomy and axillary lymph node clearance was performed: histopathology revealed a 9-mm, grade 2, oestrogen receptor-positive, progesterone receptor-negative (ER8, PR0), Her2-negative invasive ductal carcinoma, and 4/6 positive lymph nodes (T1b N2 M0). Two months later, she was readmitted with vertigo, diplopia, facial weakness, and ataxia, setting the diagnosis anti-Ri syndrome recurrence. MDT recommended mammogram and ultrasound of the left breast, which were normal. Subsequently, four months after initial discharge, she suffered another neurological recurrence; due to concomitant abdominal pain, PET-CT was performed demonstrating a hypermetabolic right ovarian focus. Bilateral salpingo-oophorectomy was performed as per gynaecology MDT and final histology showed normal tubes and ovaries. She has remained on remission since then, with a negative annual mammogram follow-up.

**Conclusions:**

In conclusion, we report a case of OMS associated with breast cancer anti-Ri onconeural antibody. Its manifestations preceded the diagnosis of malignancy and it persisted after cancer treatment, underlining the importance for high clinical suspicion in cases of classical paraneoplastic neurological syndromes as well as the need for long-term clinical follow-up.

## Background

Paraneoplastic neurological syndromes (PNS) constitute rare neurological complications of malignant disease [1, 2], manifesting in <1% of patients with cancer [3]. They are induced by an enhanced auto-immune response against neuronal self-antigens which are expressed by the tumour [3–5] and can affect any component of the nervous system [1–3]. Importantly, the implicated immune cascades are triggered in the tumour microenvironment, and are therefore unrelated to the extent of local or distant spread [5]. In the same respect, they frequently precede other clinical manifestations of the underlying malignancy, offering an opportunity for early diagnosis and treatment [3, 6].

Opsoclonus-myoclonus syndrome (OMS) presents with chaotic ocular saccades (opsoclonus), spontaneous muscular jerking (myoclonus) that may be accompanied by ataxia, strabismus, aphasia, or mutism [4]. Its paraneoplastic variant in the adult is most commonly associated with small cell lung cancer (SCLC), followed by breast cancer [7, 8]; its onset is later and its diagnosis worse compared with idiopathic OMS [7, 9]. Anti-Ri is the most commonly implicated auto-antibody, targeted against Nova-1 and Nova-2 which constitute widely expressed antigens in the central neural system (CNS); hence, when these antigens are also expressed by a tumour, an auto-immune reaction may be induced.

Hereby, we present a case of paraneoplastic OMS associated with breast cancer. Neurological manifestations preceded the diagnosis of malignancy and recurred following therapeutic surgery. Anti-Ri was the identified auto-antibody with no other abnormalities in the laboratory and radiological neurological work-up.

## Case presentation

A 43-year-old premenopausal Caucasian woman with a medical history of hypertension was admitted to her local hospital following an episode of focal seizure. A few weeks prior to admission, she developed urinary retention while at home for which she required urinary catheterization. At the time this was thought to be due to a combination of constipation and urinary tract infection. Additionally, she had developed increasing leg weakness and reported vivid auditory hallucinations with attendant insomnia.

On the day of admission, the initial focal seizure progressed to generalised tonic-clonic seizures and was she subsequently loaded with phenytoin, valproate, and levetiracetam. She was referred to the Neurology team after developing bilateral ptosis, complex gaze palsy with suspected bilateral oculomotor nerve lesions, a suspected internuclear ophthalmoplegia, and diminished reflexes, and was later intubated on the grounds of worsening respiratory fatigue with impending respiratory arrest. Following that, she underwent plasmapheresis and received high-dose steroids for a suspected diagnosis of Bieckerstaffs’s brainstem encephalitis (post-infectious ophthalmoplegia, ataxia, and lower limb areflexia). She was later transferred to our hospital, where a tracheostomy was inserted for ongoing ventilation. Initial workup included whole body contrast-enhanced computed tomography (CT) scan, viral serology [hepatitis virus A, B, and C, human immunodeficiency virus, and autoimmune serology (voltage-gated calcium channel, voltage-gated potassium channel, aquaporin 4, anti-N-methyl-D-aspartate (NMDA) receptor, IgG ganglioside-monosialic acid (GM1), nuclear Hep2, myeloperoxidase (MPO), anti-Yo, anti-Ri antibodies)]. In addition, the patient underwent magnetic resonance imaging (MRI) of the head and spine.

The CT scan revealed an enhancing right axillary lymph node, which in combination with Anti-Ri antibody positivity raised the spectre of paraneoplastic opsoclonus-myoclonus syndrome. All other tested antibodies were found negative. MRI of the head revealed subtle nonspecific white matter signal change within the centrum semiovale without any mass lesions, while MRI of the spine showed no cord lesions.

Breast surgical consultation was requested for the axillary lymph node found on CT scan. An ultrasound scan was performed which showed a right axillary lymph node with cortical thickness of 41mm which was subjected to ultrasound-guided core needle biopsy; no other focal lesion was identified in either breast. Clinical examination of the breast did not reveal any palpable abnormality, while mammography was not performed as the patient was intubated. Histology revealed poorly differentiated carcinoma with immune-phenotypical features consistent with primary breast malignancy. The case was subsequently discussed at a multi-disciplinary team (MDT) meeting where a decision was made to proceed with right mastectomy and axillary clearance after staging with positron emission tomography (PET)/CT in order to exclude any distant metastatic disease. PET/CT was negative and therefore a right mastectomy and axillary lymph node clearance was performed without any complications.

Following this, the patient made a remarkable recovery. Her tracheostomy was removed 10 days later and her swallowing function, which was initially weak requiring nasogastric feeding, recovered steadily in the weeks following her surgery. She reported ongoing dizziness despite the recovery of her eye movement disorder. It was thought that this was primarily due to vestibular deconditioning. She remained safe while mobilising on the ward with no falls and no determined need for mobility aids. She was discharged home with antiepileptic medications (clonazepam, prochlorperazine) as well as on a steroid tapering regimen.

Final histopathology revealed a 9-mm, grade 2, oestrogen receptor-positive, progesterone receptor-negative (ER8, PR0), Her2-negative invasive ductal carcinoma in the right upper outer quadrant, and 4/6 positive lymph nodes (CK7+/CK20−, AE1/AE3+; T1b N2 M0). She received adjuvant endocrine treatment with tamoxifen, adjuvant chest wall and supraclavicular fossa radiotherapy, as well as 4 cycles of epirubicin-cyclophosphamide (EC)-paclitaxel which was well tolerated.

Two months later she was admitted again due to near permanent vertigo, intermittent diplopia, facial weakness, and ataxia. She was clinically diagnosed with a recurrence of her anti-Ri syndrome. MDT recommended mammogram and ultrasound of the left breast which was normal. Subsequently, four months after initial discharge, she suffered another neurological recurrence; due to concomitant abdominal pain, PET-CT was performed demonstrating a hypermetabolic right ovarian focus. Bilateral salpingo-oophorectomy was performed as per gynaecology MDT and final histology showed normal tubes and ovaries. She has remained on remission since then, with a negative annual mammogram follow-up.

## Discussion and conclusions

The original definition of PNS included any central or peripheral nervous system, neuromuscular junction, or muscular syndrome of unknown cause that is associated with malignant disease [1, 2]. This vague description did not specify the causal relation between the neurological and cancerous disease. However, the autoimmune nature of these disorders has now been demonstrated: they are mediated by antibodies targeted against tumour-produced antigens that are normally expressed by native neural cells (onconeural antibodies) [10, 11]. Such antibodies are detected in 70–80% of PNS cases [12]; therefore, their presence is not compulsory for the diagnosis of a paraneoplastic syndrome, while PNS may be diagnosed in the absence of a known primary cancer [13]. In more detail, PNS are subclassified in classical, when their association with malignant disease is well established, and non-classical [13]. Similarly, onconeural antibodies whose association with cancer has been well-defined are classified as well-characterised and include anti-Ho, Yo, CV2, Ri, Ma2, and amphiphysin [13]. A definite PNS may be diagnosed in the following settings: (a) cancer developing within five years of diagnosing a classical neurological syndrome; (b) a non-classical syndrome that resolves or improves after cancer treatment without concomitant immunosuppressive therapy; (c) a non-classical syndrome with associated onconeural antibodies and cancer developing within 5 years of the former; or (d) a neurological syndrome associated with a well-characterised onconeural antibody.

Breast cancer has been associated with numerous immunogenic cascades [14]. More specifically, functional loss or mutations of the p53 tumour suppressor gene may result in unchecked cell division with an attendant production of mutated proteins that prime the immune system to recognise tumour-expressed epitopes as allogeneic. DNA repair defects, such as those resulting from BRCA 1/2 mutations may have similar effects. These novel antigens are drained through the lymphatic system and can incite the selection and expansion of specific B and T lymphocyte series [4]. However, these tumour-associated antigens may belong to a family of commonly shared self-antigens [5]. This amplified immune response, coupled with impaired auto-recognition, culminates in autoimmune-mediated damage [4]. Importantly, these triggering events take place in the tumour microenvironment, and are therefore not influenced by local or distant spread [5]; positive hormone receptor expression, on the other hand, has been associated with a higher propensity for autoimmune phenomena [5]. OMS, sensory and motor-type neuropathies, cerebellar degeneration, stiff-person syndrome, retinopathy, and encephalomyelitis are included among the most frequent breast cancer-associated paraneoplastic neurological syndromes [4].

Paraneoplastic OMS is a rare manifestation of cancer presenting with chaotic, synchronous ocular saccades (opsoclonus), spontaneous, brief muscular jerking (myoclonus) that may be accompanied by ataxia, strabismus, aphasia, or mutism [4]. In the adults, it is most commonly associated with SCLC and breast cancer [7, 8]; neuroblastoma is the most common association in the paediatric population [7, 8]. Ellenberg et al. were the first to report its diagnosis in a breast cancer patient [15], while Royal et al. first identified an attendant anti-neural autoantibody [16]. In the largest related case-series from a large academic surgical centre, Murphy et al. reported only 5 cases of anti-Ri-associated breast cancer PNS among 17,725 patients treated over a period of 20 years, only one of which developed OMS [6]. Anti-Ri is the most commonly implicated culprit, by targeting Nova-1 and Nova-2, two widely expressed antigens in the CNS, with anti-Yo and anti-Hu being less frequently identified [4].

Compared with idiopathic OMS, its onset is later and carries a worse prognosis [7, 9]. Neurological symptoms usually precede the diagnosis of breast cancer [6], but further paraneoplastic OMS progression is unpredictable: it may spontaneously resolve or variably respond to treatment [11]. Intriguingly, despite the fact that the underlying autoimmune process is instigated in the tumour microenvironment [5], the syndrome may persist following tumour resection [17]. Despite the lack of any pathognomonic pathological changes [11], Purkinje cell loss has been identified in previous cases of paraneoplastic OMS [4]; its persistence may explain the tendency of the syndrome to relapse after surgical resection, similar to other paraneoplastic diseases with permanent neurological damage [11]. This mandates the timely initiation of immune suppressive therapy independently of surgical scheduling, as well as its continuation in accordance to the clinical response of the patient, in addition to tumour-specific treatment [6, 17, 18]. Regardless of the potential of immune suppressive treatment to induce partial or complete recovery [18], neurological complications have been reported as the most common cause of death [4]. Moreover, symptomatic or biochemical relapse in the form of antibody positivity may be a signal for cancer recurrence and necessitates appropriate clinical investigation [6, 19].

Consistent with the above discussed typical paraneoplastic OMS natural history, neurological symptoms manifested before the diagnosis of breast cancer in our patient. Crucially, the presence of a neurological syndrome with a concomitant well-characterised onconeural antibody fulfilled the criteria of definite PNS, mandating a thorough work-up for the identification of the underlying malignant disease. Furthermore, despite the initial response to immune suppressive treatment and the successful breast cancer resection, our patient has suffered from neurological relapses, highlighting the importance of long-term vigilance.

In conclusion, we report a very rare case of OMS associated with breast cancer anti-Ri onconeural antibody. Its manifestations preceded the diagnosis of malignancy and it persisted after cancer treatment, underlining the importance for high clinical suspicion in cases of classical paraneoplastic neurological syndromes, as well as the need for long-term clinical follow-up.

## Data Availability

The data that support the findings of this study are available on reasonable request from the corresponding author. The data are not publicly available due to privacy or ethical restrictions.

## Abbreviations

CNS: Central neural system
CT: Computed tomography
EC: Epirubicin-cyclophosphamide
ER: Oestrogen receptor
GM1: Ganglioside-monosialic acid
MDT: Multi-disciplinary team
MPO: Myeloperoxidase
MRI: Magnetic resonance imaging
NMDA: N-methyl-D-aspartate
OMS: Opsoclonus-myoclonus syndrome
PET: Positron emission tomography
PNS: Paraneoplastic neurological syndromes
PR: Progesterone receptor
SCLC: Small cell lung cancer
